# Congenital Mandibular Hypoplasia: Patient-Specific Total Joint Replacement as a Line Extension in the Treatment of Complex Craniofacial Anomalies

**DOI:** 10.1007/s12663-022-01780-9

**Published:** 2022-09-03

**Authors:** Rüdiger M. Zimmerer, Anna Katharina Sander, Annika Schönfeld, Bernd Lethaus, Nils-Claudius Gellrich, Michael-Tobias Neuhaus

**Affiliations:** 1 Department of Oral and Maxillofacial Surgery, Leipzig University Hospital, Liebigstraße 12, 04103 Leipzig, Germany; 2 Department of Oral and Maxillofacial Surgery, Hannover Medical School, Carl-Neuberg-Str. 1, 30625 Hannover, Germany

**Keywords:** Congenital mandibular hypoplasia, Mandibular reconstruction, Temporomandibular joint, TMJ, Total joint replacement, TJR

## Abstract

**Introduction:**

Congenital mandibular hypoplasia (CMH) remains challenging because of the underlying combined hard and soft tissue deficiency. Treatment options include craniofacial distraction, orthognathic surgery, and autologous grafts, although the latter produces inadequate results after distraction and autologous grafting. Unsatisfactory long-term stability may cause relapse, necessitating reoperation.

**Material and Methods:**

We investigated the feasibility of using alloplastic total joint replacement (TJR) in growing and young adult CMH patients. The primary outcome was long-term reconstruction stability, without implant failure. Secondary outcomes were TMJ function and pain, and jaw movements achieved during surgery.

**Results:**

Three patients (age: 9–22 years) were treated by the same surgeon at one institution during 2018–2021. Anamnesis and clinical parameters were obtained from patient records. Preoperative 3D-scans were superimposed with postoperative 3D-scans and preoperative plans, including TJR-implant STL files, to measure jaw movement. All patients underwent prior reconstructive surgery. Mandibular movement of 16.4–20.1 mm in the sagittal direction was achieved. Post-TJR follow-up ranged from 24 to 42 months. No long-term complications occurred. At the latest follow-up, the maximal interincisal opening was between 21 and 40 mm, and all implants were functioning, without failure.

**Conclusion:**

In selected CMH cases, alloplastic TJR can deliver satisfactory medium-term results with predictable and stable outcomes, even in growing patients.

## Introduction

**Fig. 1:**
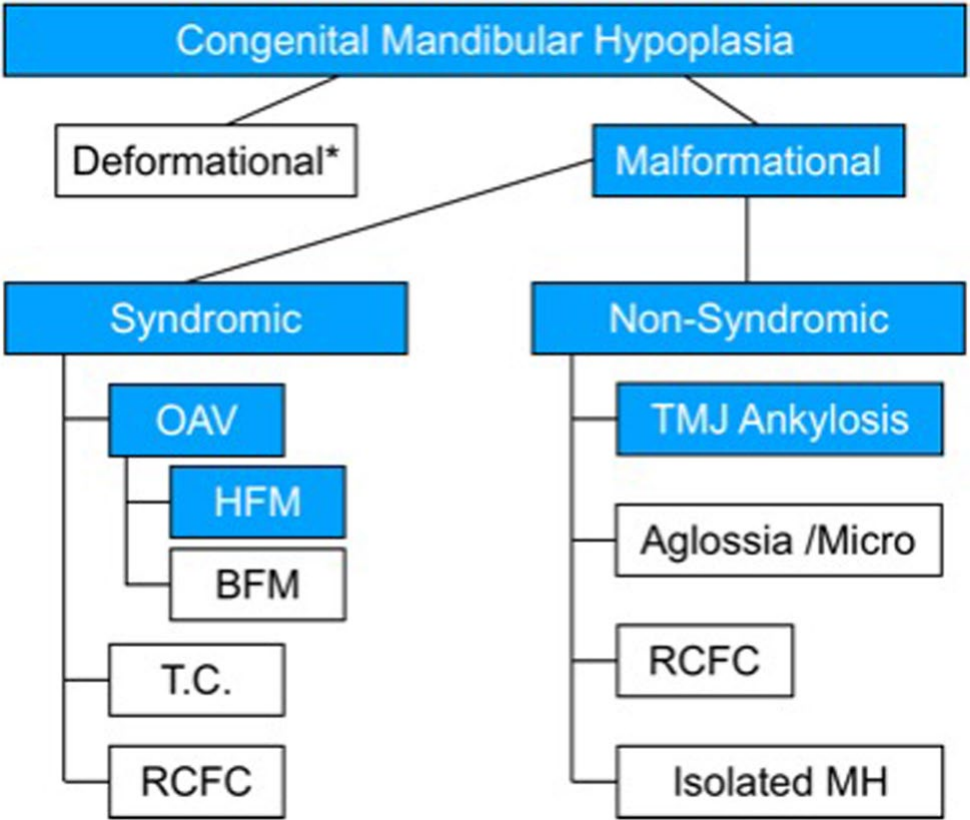
Modified according to Davinder J. Singh, MD, Scott P. Bartlett, MD: Congenital Mandibular Hypoplasia: Analysis And Classification; THE JOURNAL OF CRANIOFACIAL SURGERY/VOLUME 16, NUMBER 2 March 2005; MH, mandibular hypoplasia; OAV, oculo-auriculo-vertebral; TMJ, temporomandibular joint; HFM, hemifacial macrosomia; BFM, bilateral facial macrosomia; T.C., Treacher Collins; RCFC, rare craniofacial clefts, *Deformational MH can also be subdivided into a syndromic and non-syndromic group, to which the Pierre Robin Sequence can be assigned to according to Singh and Bartlett et al.

Mandibular hypoplasia is one of the main anomalies of the mandible and can be classified as a congenital, developmental, or acquired deformity [1]. Developmental mandibular hypoplasia is defined as the underdevelopment of the mandible for unknown reasons. These patients usually present with Class II malocclusion [1]. Acquired mandibular hypoplasia includes oncological defects, radiation damage, trauma, and hemifacial atrophy [2, 3]. Congenital mandibular hypoplasia (CMH) most frequently results from the maldevelopment of the first and second branchial arches, and can occur unilaterally or bilaterally [1]. According to a classification algorithm suggested by Singh and Bartlett [1], CMH can be further subdivided into malformational and deformational groups, which include syndromic and non-syndromic patients (Fig. 1). Congenital deformities of the mandible constitute a heterogeneous group of rare disorders with variable clinical presentations, as reflected by the inconsistent medical terms used in the literature. Most patients with CMH have associated syndromes [1]. More than 60 syndromes are associated with mandibular hypoplasia, including Goldenhar’s syndrome [3], Treacher Collins syndrome [4], Nager's syndrome [5], and the Pierre Robin sequence [4, 6]. In addition, CMH can occur in the absence of known syndromes in 6.8% of all CMH patients [1]. Goldenhar’s syndrome is part of the oculo-auriculo-vertebral (OAV) spectrum, which also encompasses hemifacial microsomia. OAV, hemifacial microsomia, and Goldenhar’s syndrome involve the eyes, ears, and spine. Although Goldenhar’s syndrome is often referred to as being hemifacial, it is bilateral (bifacial) in 10–30% of cases. However, there is usually only one dominant side. Goldenhar's syndrome may be a more complicated version of OAV, while hemifacial microsomia may be a milder version. The next most common is the mandibulofacial dysostosis group, or Treacher Collins syndrome [7].

### Clinical Challenges

Most commonly, CMH is bilateral, although the underlying deformity may be unilateral. Depending on the underlying syndrome, the midface and cranium can also be affected primarily, or as a secondary compensatory growth change on the unaffected side. Depending on the extent of hypoplasia, usually in bilateral disorders, a symmetrical profound effect on the patient’s airways may require endotracheal intubation or tracheostomy [8]. In unilateral mandibular hypoplasia, the midface and cranium are typically co-affected [3, 9, 10]. Differentiation between symmetrical and asymmetrical deformities may have a marked impact on the therapeutic algorithm [11]. In terms of hemifacial CMH, the severity is graded with a focus on the ramus–condyle unit, and three-dimensional (3D) analysis is performed to characterize the mandibular phenotype under these conditions [12–14].

Either the primary deformity itself or the secondary impact of isolated unilateral mandibular hypoplasia on the growth of adjacent anatomical units leads to complex asymmetric deformities, with compensatory dental effects. In addition to the underdeveloped hard tissues, including the mandible, temporomandibular joint (TMJ), midface and cranium, the soft tissue envelope is even more deficient, with scarring and poor vascularization. Congenital soft tissue deficiency is the real clinical challenge that needs to be addressed to achieve predictable long-term success.

### Treatment Options

The treatment of CMH varies across institutions and healthcare systems and is highly dependent on the extent of the deformity and associated clinical symptoms [15]. All treatment options aim to create a symmetrical, harmonious facial appearance with stable occlusion and adequate TMJ function. Patients with OAV and those with Treacher Collins syndrome have been well studied, with numerous publications addressing their presentation and treatment, including craniofacial distraction, orthognathic surgery combined with orthodontic treatment, as well as autologous (bone) grafts or microvascular transplants [16–20]. In recent reports, distraction alone often did not solve the problem, because distraction had to be repeated, followed by orthognathic surgery and genioplasty or bone grafting procedures [16, 18, 19, 21]. However, unsatisfactory long-term results have been reported because of relapse after distraction and unpredictable resorption or growth of the costochondral graft [3, 18]. Mandibular distraction in children without respiratory or feeding difficulties remains controversial in terms of long-term mandibular growth outcomes and reduction of surgical burden [20]. Microsurgical constructions are reserved for children with large, complex mandibular defects when other options are unavailable or have been exhausted [22]. Most current treatments focus on correcting bony malformations, but none address soft tissue deficiency [15].

This study aimed to demonstrate the feasibility of alloplastic reconstruction with patient-specific total joint replacement (TJR) of the TMJ in adult and juvenile patients with CMH. The primary outcome was long-term stability of the reconstruction, without any implant failure. The secondary outcome was TMJ function and pain, as well as jaw movements achieved during surgery.

## Materials and Methods

The database of the Department of Oral and Maxillofacial Surgery, Hannover Medical School, Hannover, Germany, was screened for patients with CMH who had undergone TJR of the TMJ. Patient records were reviewed for clinical presentation, surgical details, etiology, genetic diagnosis, and classification [23, 24]. Radiological images and virtual surgical planning (VSP) data were collected and analyzed.

This study was approved by the local ethics review committee (Hannover Medical School; study no.: 9275_BO_K_2020).

### TMJ Prosthesis and Virtual Surgical Planning

**Fig. 2:**
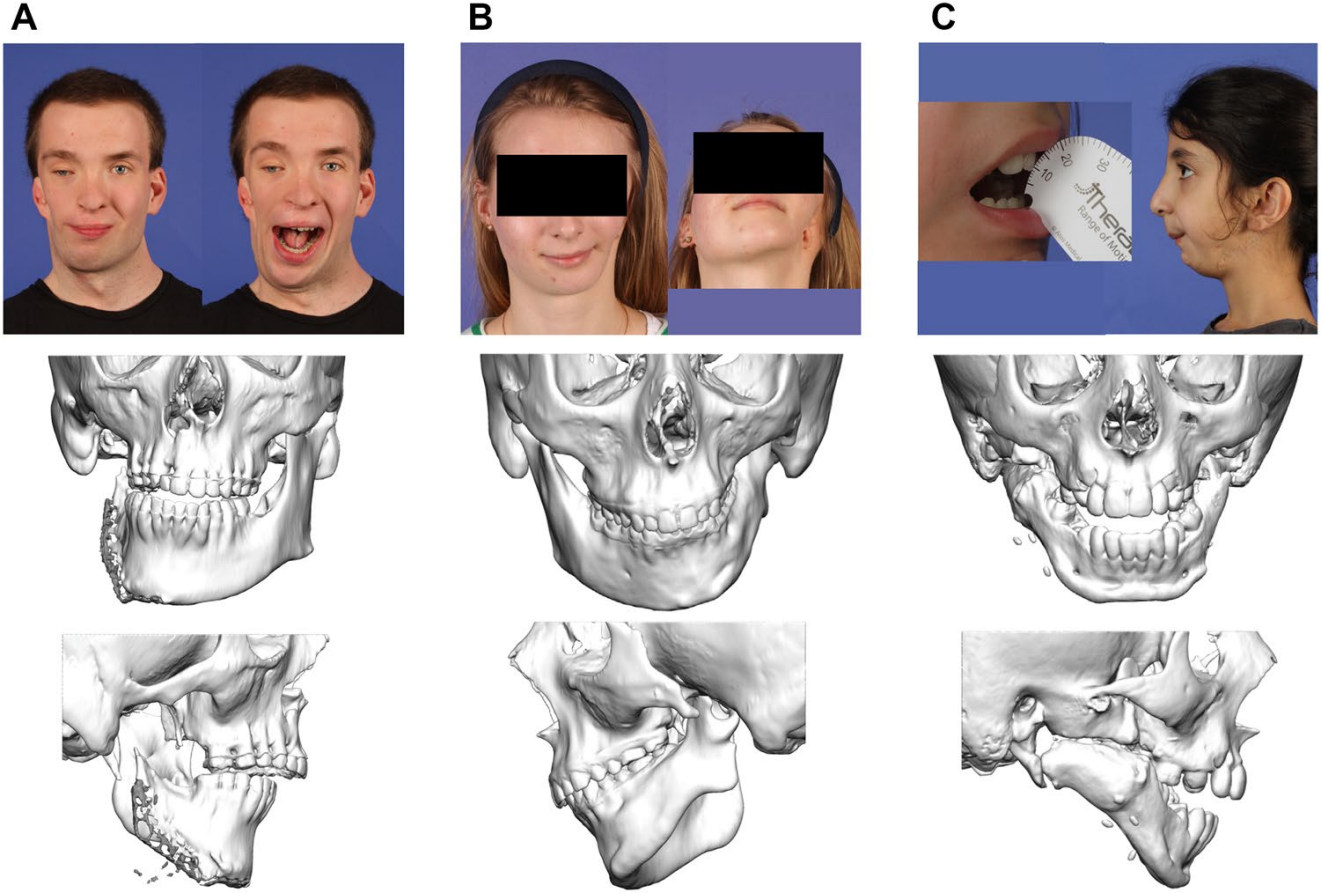
Case 1–3 preoperatively, photography and 3D-rendering of the skull and mandible. Showing each’s primary deformity **A** Case 1, a 20-year-old man with Goldenhar syndrome. Pruzansky III, O_2_ M_3_ E_2_ N_1_ S_2_, previous autologous reconstruction with fibular free flap. **B** Case 2, 22-year-old women with Goldenhar syndrome. Pruzansky IIb, O_0_ M_2b_ E_3_ N_0_ S_3_, previous mandibular distraction. **C** Case 3, 9-year-old girl, with bilateral mandibular hypoplasia, MIO reduced to 10 mm, severe soft tissue deficiency, previous surgeries: costochondral graft, mandibular distraction, interpositional gap arthroplasty

**Fig. 3:**
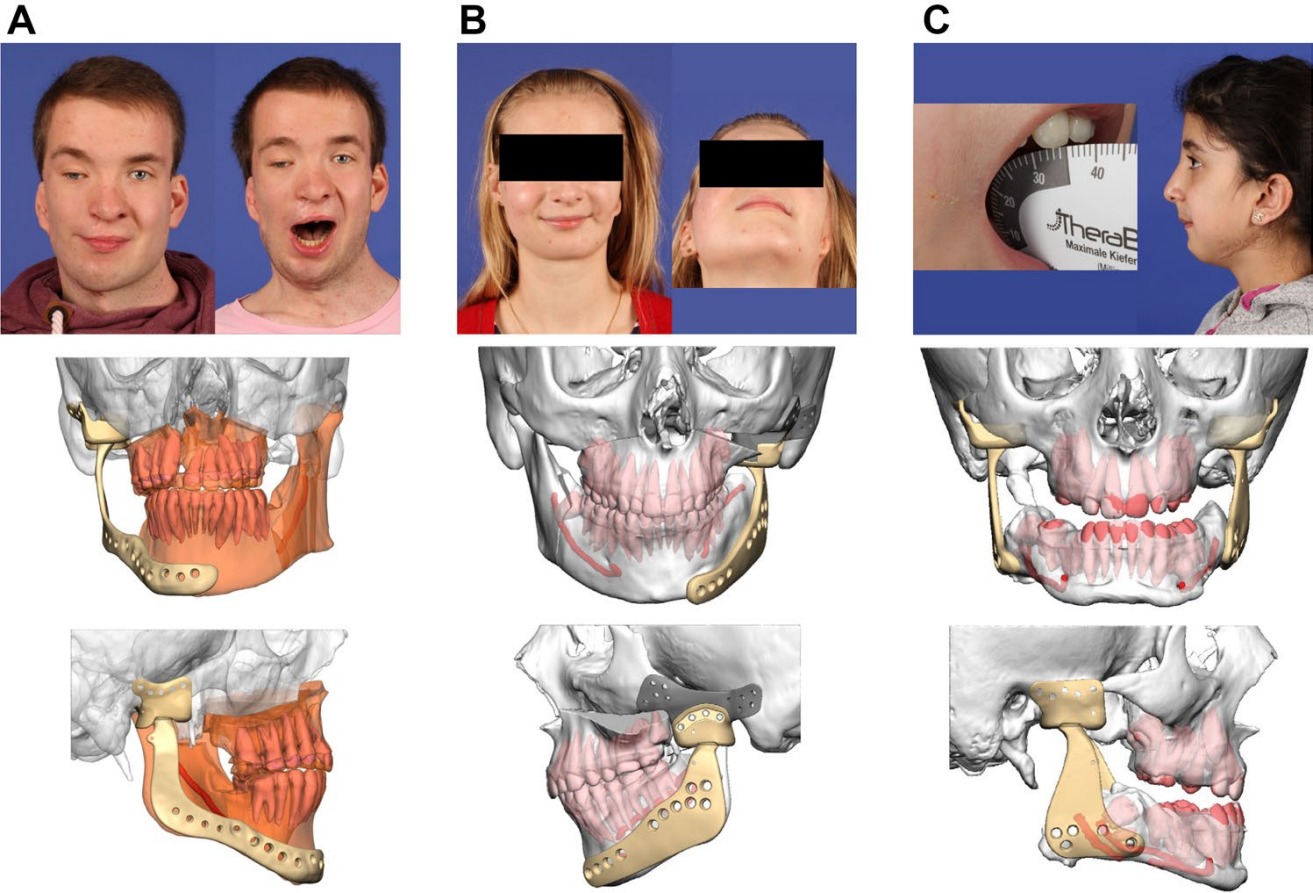
Case 1–3 postoperatively, photography and virtual surgical planning with TMJ implants. **A** Case 1, unilateral TJR on the right side with extended mandibular component due to mandibular defect, with additional LeFort I osteotomy and contralateral SSO (sagittal split osteotomy). Dentition and inferior alveolar nerve visualized. **B** Case 2, unilateral TJR on the left side with extended fossa component, due to complete aplasia of the zygomatic arch. Additional LeFort I osteotomy and contralateral SSO. Dentition and inferior alveolar nerve visualized. **C** Case 3, bilateral TJR with anterior movement of the mandible, class I dentition with head bite planned in anticipation of maxillary growth. Dentition and inferior alveolar nerve visualized

Patient-matched temporo-mandibular total joint prostheses were obtained from Zimmer Biomet (Jacksonville, Florida, USA). As we previously described, during VSP, the prostheses were equipped with functional design features, including an anterior suture hole in the mandibular and fossa components to prevent condylar sagging, and flanges at the posterior aspect of the mandibular component to identify the one-fit-only position [25]. One patient required an extended mandibular component due to resorbed bony reconstruction, resulting in a hemi-mandibular segmental defect on the right side (Figs. 2A, 3A). Another case required an extended fossa component with a titanium base plate due to complete aplasia of the zygomatic arch and glenoid fossa (Figs. 2B, 3B). The fossa was made of ultra-high-molecular-weight polyethylene and the mandibular components were made of titanium instead of cobalt–chrome–molybdenum.

### Intraoperative Real-Time Navigation and Intraoperative 3D Imaging

The benefits of intraoperative real-time navigation and 3D imaging in the complex reconstruction of the TMJ have been demonstrated by our group [25, 26]. As previously described, the initial planning computed tomography (CT) datasets were used for navigation. The patients were registered using the intermaxillary navigational splint technique. The STL files of the patient-matched prostheses were uploaded to the 3D planning platform (Brainlab Elements®, Brainlab, Munich, Germany). Using the Brainlab trajectorial planning option, the drilling vectors and lengths for fossa component fixation were virtually determined. Navigation was used during dissection and resection of the medial skull base, during drilling, and for final implant position control [25, 26]. Following the implantation of single components, intraoperative 3D cone-beam computed tomography (CBCT) was performed. The acquired dataset was fused with the preoperative VPS intraoperatively to visualize the precision of the reconstruction.

### 3D-Image Analysis

**Fig. 4:**
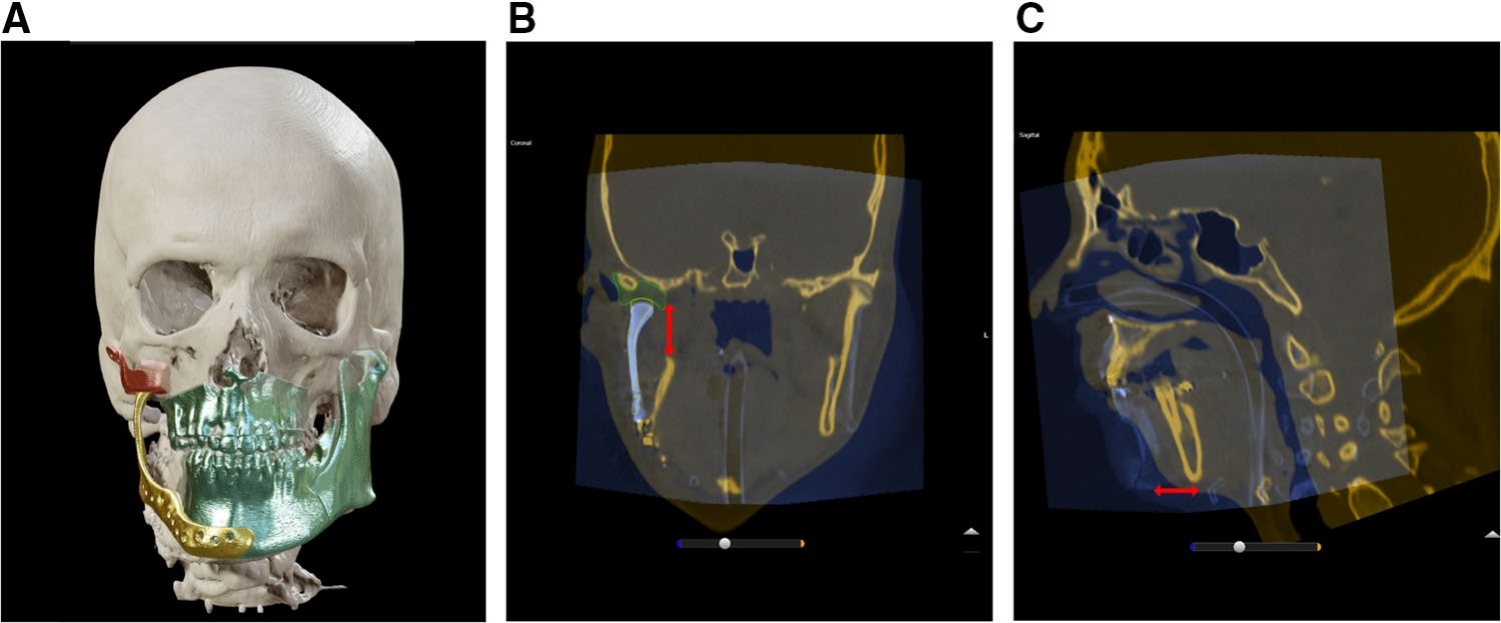
Case 1 **A** 3D-rendering (Brainlab® Elements, Brainlab, Munich, Germany) of preoperative CT-scan with TJR fossa (red) and mandible (gold) component. Simulated movements of the maxilla and mandible body are shown in green, with contralateral sagittal split osteotomy. **B** Fusion of preoperative (amber) and postoperative (blue) CT or CBCT scan, coronal orientation. Planned positions of TJR fossa (green) and mandible (yellow) component are shown and match perfectly to achieved (blue) position. Red arrow indicates gained height of mandibular ramus (20.2 mm). **C** Fusion of preoperative (amber) and postoperative (blue) CT or CBCT scan, sagittal orientation. Red arrow indicates sagittal anterior movement of the mandible/pogonion (16.4 mm)

As previously described, pre-, postoperative and follow-up 3D-images were fused using BrainLab Elements (BrainLab Elements). STL files of the prostheses, such as fossa and mandibular components, were provided by the manufacturer (Zimmer Biomet, Jacksonville, Florida, USA) and uploaded into the initial planning computed tomography data set (Fig. 4). The postoperative positions of the components were compared with intraoperative and follow-up 3D-images. In addition, distances of surgically achieved mandibular and maxillary movements were measured in the postoperative and follow-up 3D-images (Fig. 4B, C).

### Retrospective Evaluation of the Postoperative Follow-Up Visits

According to our department’s standard protocol, patients who have undergone TJR are usually followed up at 1-, 3-, 6-, and 12-months postoperatively. Once the first year was completed, the annual follow-up interval was determined. The standard follow-up visits included radiological and clinical examinations. Radiological examinations included panoramic radiography or CBCT imaging. The standard-of-care clinical examinations included monitoring the range of mandibular movements, facial nerve function, pain, and occlusion.

## Results

### Patients

In total, three patients were included in this retrospective analysis. The demographic details and anamnesis of the enrolled patients are summarized in Table 1. The individual patient history revealed that all patients had been treated previously with distraction, bone grafting, free-flap reconstruction, or arthroplasty. The presented alloplastic TJR was thus performed as a secondary or tertiary TMJ reconstruction after failed conventional treatments or relapse (Fig. 2A, B). Surgical interventions were performed between 2017 and 2020. Two patients were diagnosed with Goldenhar’s syndrome, and one patient showed non-syndromic bilateral CMH (Fig. 2, Table 1). Figure 2 shows the enrolled patients and each individual’s dominant deformity. Both Goldenhar’s syndrome patients were classified according to the OMENS + and Pruzansky–Kaban classifications (Fig. 2, Table 1).

**Table 1:** Patients

	Case		
	1	2	3
Gender	Male	Female	Female
Age at surgery	20	22	9
Congenital mandibular hypoplasia (CMH)	Syndromic	Syndromic	Non-syndromic
Dominant side	R	L	Bilateral
Type of syndrome	Goldenhar	Goldenhar	–
Pruzansky classification	III	IIb	–
OMENS + Classification	O_2_ M_3_ E_2_ N_1_ S_2_	O_0_ M_2b_ E_3_ N_0_ S_3_	Severe soft tissue deficiency
Additional surgery	LeFort 1 & SSO	LeFort 1 & SSO	–
Preop. orthodontic treatment	+	+	
Postop. orthodontic treatment	+	+	+
Previous surgeries	Fibula free flap	Unilateral mandibular distraction	CCG, bilateral mandibular distraction, IGA

OMENS+: O, orbital distortion; M, mandibular hypoplasia; E, ear anomaly; N, nerve involvement; S, soft tissue deficiency; SSO, sagittal split osteotomy; CCG, costochondral graft; IGA, interpositional gap arthroplasty (fat)

### Postoperative Measurement of Movements

Surgically achieved movements of the maxilla and mandible were measured using the Brainlab Elements 3D planning platform (Brainlab, Munich, Germany) (Fig. 3, Table 2). The most significant movements (ranging between 16.4 and 20.1 mm) were observed in the mandible in the sagittal projection. The height of the vertical rami, gained through TJR, was between 13.4 and 24.4 mm. In the two cases of Goldenhar´s syndrome, the occlusal cant could be sufficiently reduced and levelled to the horizontal plane (Table 2). Condylar sagging of the mandibular component was observed and ranged between 0.1 and 10.4 mm.

**Table 2:** Surgical movements

	Case		
	1	2	3
Occlusal cant preop [°]	18.2	7.1	3
Occlusal cant postop [°]	9.3	3.7	3
Upper jaw sagittal 11 [mm]	4.9	3.1	0
Pogonion sagittal [mm]	16.4	18.8	20.1
Pogonion lateral [mm]	9.2	24	2.6
Vertical ramus height [mm]	20.2	13.4	20.7
Vertical ramus height 2nd side [mm]			24.4
Condylar sagging [mm]	4.8	10.4	0.1/5.4

### Follow-up

The follow-up period of the three patients ranged from 24 to 42 months. At the latest follow-up, all prostheses were functional. No revision surgeries were required, and no material failures of the prosthesis components were observed. Finally, the virtually planned outcome positions of the maxilla and mandible remained stable over time and no relapses were identified. This also applied to occlusion (Fig. 3). Long-term complications, such as permanent facial nerve palsy, chronic infections, pain, or discomfort, did not occur (Table 3). The maximal interincisal opening was stable at 21 to 40 mm over time at the follow-up visits. All patients were fed a full diet.

**Table 3:** Adverse events

	Case		
	1	2	3
*Complications*			
Facial nerve palsy			
Transient (< 6 month)			
Frontal branch	+	+	+
Buccal branch		+	
Marginal branch		+	
Permanent (> 6 month)	–	–	–
Others (SSI*, PJI**, material failure, scars, allergies, discomfort, salivary fistula)	–	–	Keloid
*TMJ function & pain*			
TMJ Pain [VAS]	0	0	1
MIO [mm]	21	34	40
Mediotrusion (TJR side) [mm]	1	2	0
Protrusion [mm]	3	2	2

*MIO* maximal interincisal opening, *TMJ* temporomandibular joint, *TJR* total joint replacement

*Surgical site infection

**Prosthetic joint infection

## Discussion

In this study, we used alloplastic TJR for reconstruction of congenital mandibular deformities in growing patients with CMH. We achieved mandibular movement of 16.4–20.1 mm in the sagittal direction. Over the follow-up period of 24–42 months, no long-term complications occurred. The maximal interincisal opening achieved at the last follow-up was 31.67 ± 7.93 mm, and all implants were functional.

The application of alloplastic TJR in the construction of congenital mandibular deformities is rare, and only a few case reports have been published to date, mostly focused on non-growing patients [27–31]. The treatment of patients with alloplastic TJR in general, in adults, and in juvenile patients differs markedly across countries and healthcare systems. Nevertheless, this approach is increasingly used worldwide, since VSP and CAD/CAM techniques have been introduced and allowing for patient-specific prosthesis design [25, 31]. Patient-matched prostheses are not only used because of their perfect anatomical fit to challenging anatomical sites, but mainly because it is possible to implement virtually planned mandibular movements in their design and combine them with conventional (and bimaxillary) orthognathic surgery [29, 30, 32–34]. In conventional orthognathic surgery, a reliable TMJ is needed to ensure long-term skeletal and occlusional success. This is absent in cases with congenital TMJ deformities. Thus, TJR can be considered a line extension of orthognathic surgery, in cases with severe TMJ deformities.

Our findings demonstrated the feasibility of patient-matched TJR combined with orthognathic surgery in the treatment of CMH, even in multiply pretreated growing patients. Follow-up in the medium term showed adequate TMJ function with a corrected and symmetrized stable skeletal and occlusional situation that could not be achieved by previous autologous reconstruction or distraction osteogenesis. In addition, it facilitates extensive sagittal mandibular movements without a skeletal relapse. Furthermore, TJR can be used to increase the vertical ramus height and camouflage lost facial prominences, such as the mandibular angle.

### Reconsideration of Distraction Osteogenesis and Costochondral Grafting

There is a common misconception that in distraction osteogenesis and costochondral grafting in CMH cases, deficient bone needs to be replaced by distraction or bone alone, in order to correct the deformity. However, neither distraction nor bone grafting, including costochondral grafts, can create a stable soft tissue envelope that undergoes hyperplasia and provides significant improvement, as they focus on re-establishing the bony deficiency alone. After grafting or distraction, the soft tissue envelope is temporarily stretched, but the deficiency and scarring again lead to retraction of the soft tissue envelope in the long term. This results in relapse of the distraction and graft resorption. Consequently, there are currently only two options: firstly, to use rigid, non-resorbable materials that are strong enough to resist soft tissue retraction and maintain the surgical outcome in the long term and, secondly, to improve the soft tissue envelope prior to or simultaneously with the grafting procedure or distraction.

### Surgical Risks

The list of potential complications associated with costochondral grafts is extensive. In addition to inducing pneumothorax, the graft must be placed in a nonexistent or rudimentary glenoid fossae. The graft also has to be attached to a rudimentary or severely malformed ramus, usually in areas with scarring, poor vascularization, and a deficient soft tissue envelope, particularly in severe forms of hemifacial macrosomia. Some authors have reported that the orientation of the rib at the graft site during surgery might be challenging, and that the graft dislodged laterally or superiorly. Most importantly, costochondral grafts have shown unpredictable growth and may fracture in the costochondral joint. Finally, there is the risk of graft infection, resorption, pain, relapse, and facial nerve damage [35]. Moreover, the risk of temporary or permanent facial nerve palsy is not negligible in cases with complex congenital deformities, massive ankylosis, and multiple operated joints, independent of implanted materials.

### Alloplastic Total Joint Replacement

Although alloplastic TJR does not increase the quantity and quality of deficient soft tissues, the prosthesis is sufficiently rigid to preserve the steady state of a stretched soft tissue envelope. A few case reports of TJR in congenital deformities have been published and showed promising results [29–31]. The surgical risk of TJR is comparable to that of any autologous procedure or distraction, since the same surgical approaches are used. Even if the risk for rib grafts is thought to be low, TJR carries no risk of donor-side morbidity, and there are no bone grafts to be lost or resorbed. However, alloplastic materials can also become infected, and periprosthetic joint infections (PJIs) are difficult to treat. Allergic reactions to implant materials can also occur (cobalt, chromium, molybdenum, nickel, and polyethylene). Moreover, 8–12 weeks are required for the manufacturing of the prosthesis.

### Effect on Skeletal Growth and Facial Asymmetry

**Fig. 5:**
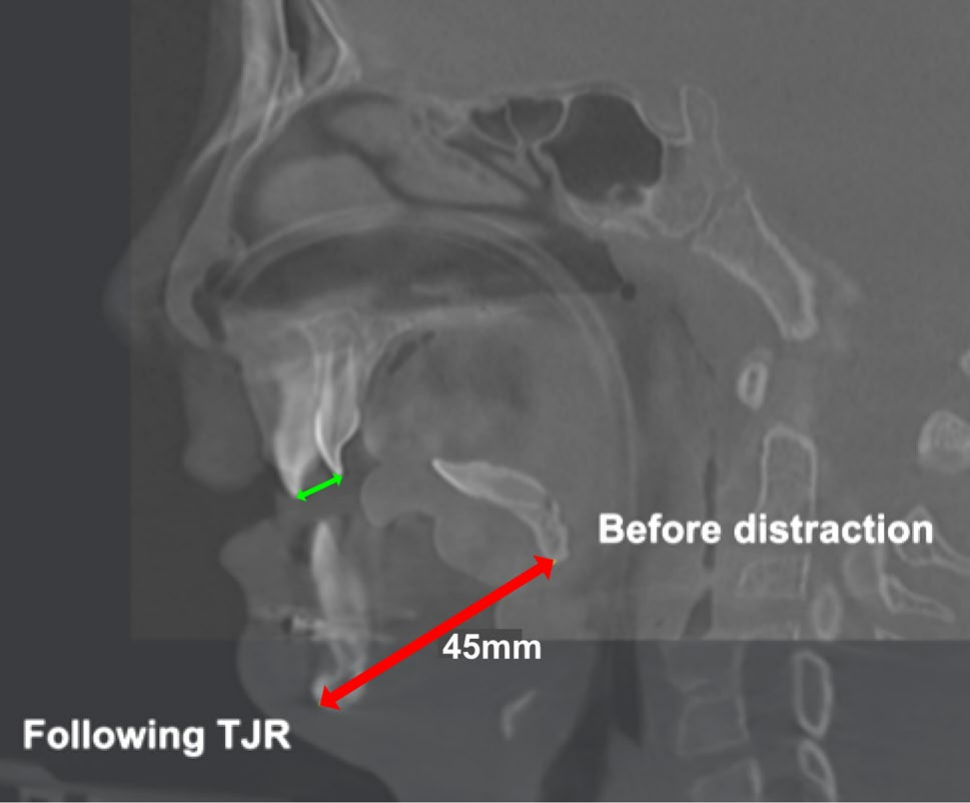
Case 3—Fusion of initial/pre-distraction (surgery prior to TJR) CBCT-scan and postoperative/post-TJR CBCT-scan. Patient suffered from severe bilateral mandibular hypoplasia with permanent tracheotomy and being unable to eat. Red arrow indicates large distance mandibular movement between initial situation and situation after TJR (45 mm). Movement between post-distraction and post-TJR situation was 20.1 mm (Table 2). Green arrow indicates maxillary growth between both CBCT-scans

Alloplastic materials do not have any growth potential, but they can deliver predictable short- and long-term clinical situations. Costochondral grafts have an inherent growth potential but are unpredictable [15]. Long-term reports of mandibular growth in children who underwent reconstruction with costochondral grafts showed that excessive growth occurred on the treated side in 54% [27, 28, 36–40]. An investigation of mandibular growth after costochondral grafting supported previous experiments regarding the inability of the graft to adapt to the growth velocity of the new environment. Furthermore, no mandibular growth can be expected on the affected side of patients with CMH, particularly those with Pruzansky III, where no TMJ is present. In the present study, we also showed that maxillary growth in a 9-year-old female with ankylosis due to failed costochondral grafts and a relapse following distraction and conventional interpositional gap arthroplasty (case No. 2), was not affected by bilateral TJR (Fig. 5). LG Mercuri stated, that *“These patients could be better off undergoing alloplastic TMJ TJR, knowing that revision and/or replacement surgery may likely be required in the future depending on growth, rather than incurring continued failures with autogenous tissues that will also very likely require further surgical intervention in the future”* [27].

The long-term outcomes of TJR of the TMJ in patients with CMH are promising. The literature shows a > 90% success rate after 20 years of TJR in general [41–43]. Furthermore, prosthesis exchange to material wear can be ignored, because even the replaced TMJ is considered to be a non-load-bearing joint. Friction wear is unlikely to occur during functional use. However, this needs to be proven by long-term follow-up studies of these patients and materials. CMH is a rare disease, that’s why our study is limited by its small sample size. The number of CMH patients treated with our presented technique and the follow-up period have to be increased in future studies.

## Conclusion

In selected cases with CMH, including those who have undergone multiple previous treatments, TJR of the TMJ combined with orthognathic surgery provides a predictable and medium-term stable treatment option, even in growing patients. In future, distraction osteogenesis and autologous grafting procedures should be critically evaluated for each patient individually, particularly for those with severe soft tissue deficiency (OMEN**S**_**2-3**_).
